# *Salmonella* Flagellin Activates NAIP/NLRC4 and Canonical NLRP3 Inflammasomes in Human Macrophages

**DOI:** 10.4049/jimmunol.2000382

**Published:** 2020-12-30

**Authors:** Anna M. Gram, John A. Wright, Robert J. Pickering, Nathaniel L. Lam, Lee M. Booty, Steve J. Webster, Clare E. Bryant

**Affiliations:** * Immunology Catalyst Programme, GlaxoSmithKline, Stevenage SG1 2NY, United Kingdom;; † School of Biochemistry and Immunology, Trinity Biomedical Sciences Institute, Trinity College, Dublin 2, Ireland; and; ‡ Department of Veterinary Medicine, The University of Cambridge, Cambridge CB3 OES, United Kingdom

## Abstract

Infection of human macrophages with *Salmonella enterica* serovar Typhimurium (*S.* Typhimurium) leads to inflammasome activation. Inflammasomes are multiprotein complexes facilitating caspase-1 activation and subsequent gasdermin D–mediated cell death and IL-1β and IL-18 cytokine release. The NAIP/NLRC4 inflammasome is activated by multiple bacterial protein ligands, including flagellin from the flagellum and the needle protein PrgI from the *S.* Typhimurium type III secretion system. In this study, we show that transfected ultrapure flagellin from *S*. Typhimurium induced cell death and cytokine secretion in THP-1 cells and primary human monocyte-derived macrophages. In THP-1 cells, NAIP/NLRC4 and NLRP3 played redundant roles in inflammasome activation during infection with *S.* Typhimurium. Knockout of *NAIP* or *NLRC4* in THP-1 cells revealed that flagellin, but not PrgI, now activated the NLRP3 inflammasome through a reactive oxygen species– and/or cathepsin-dependent mechanism that was independent of caspase-4/5 activity. In conclusion, our data suggest that NLRP3 can be activated by flagellin to act as a “safety net” to maintain inflammasome activation under conditions of suboptimal NAIP/NLRC4 activation, as observed in THP-1 cells, possibly explaining the redundant role of NLRP3 and NAIP/NLRC4 during *S.* Typhimurium infection.

## Key Points

- *Salmonella* infection leads to NAIP/NLRC4 and NLRP3 inflammasome activation.
- Flagellin activates the NAIP/NLRC4 and NLRP3 inflammasome in human macrophages.
- Flagellin mediates NLRP3 activation in a ROS- and/or cathepsin-dependent manner.

## Introduction

*Salmonella* species are Gram-negative bacteria causing diverse diseases, ranging in severity from mild gastroenteritis to systemic infection in humans. *Salmonella enterica* serovar Typhimurium (*S.* Typhimurium) triggers an inflammasome response in murine and human macrophages (1, 2). Inflammasomes are multiprotein complexes facilitating caspase-1 activation. Active caspase-1 cleaves the proforms of the cytokines IL-1β and IL-18 and the pore-forming protein gasdermin D (GSDMD), leading to cytokine maturation and their release from the cell through GSDMD pores (3). In human and murine macrophages, a redundant role for NAIP/NLRC4 and NLRP3 inflammasome activation has been demonstrated upon *S.* Typhimurium infection (4–7). It remains unclear, however, which ligand(s) trigger NLRP3 and the underlying mode of activation. The NAIP/NLRC4 inflammasome is well characterized in terms of its molecular activation mechanism (8). Knockout (KO) of *NLRC4* leads to loss of inflammasome activation at early time points during infection (5). In murine macrophages, NAIP/NLRC4 inflammasomes are formed upon sensing the bacterial protein ligands flagellin and the needle and/or rod of type III secretion systems (T3SS) from bacteria such as *S.* Typhimurium (9–11). Biochemical, structural, and genetic data show that the bacterial ligands are bound by individual murine Naip proteins: the needle protein is sensed by Naip1, the rod protein by Naip2, and flagellin by Naip5 and Naip6 (10, 12–16). Ligand binding activates Naip, allowing it to bind to one NLRC4 molecule, which in turn is activated and further recruits more NLRC4 molecules until an open ring-like structure is formed (17). Formation of the inflammasome platform promotes interaction with the adaptor protein ASC and procaspase-1, resulting in activation of caspase-1.

The majority of studies have focused on murine rather than human inflammasome activation in response to *S.* Typhimurium. One prominent difference between murine and human NAIP/NLRC4 inflammasomes is that humans express a single NAIP protein, which shares the highest homology with Naip1 out of all murine Naips. Human NAIP was, therefore, initially thought to sense only the needle protein (10). In addition to rod proteins from other bacterial species, the *Salmonella* rod protein PrgJ has recently been shown to activate human inflammasomes (18, 19). Conflicting data have been reported for flagellin-mediated inflammasome activation in different human macrophage models (18, 20). Primary human macrophages can sense the *S.* Typhimurium needle protein PrgI, rod protein PrgJ, and flagellin protein fliC (18, 20); however, the relative contribution of those ligands to NAIP/NLRC4 activation remains unclear. The mechanistic basis for the redundancy of NLRP3 and NLRC4 during *S.* Typhimurium infection is also not well understood.

In this study, we characterized the human inflammasome response to live *S.* Typhimurium in the PMA-differentiated macrophage-like cell line THP-1 and in primary human monocyte-derived macrophage (hMDM) and assessed the role of different NAIP/NLCR4 ligands using bacterial strains lacking flagellin or T3SS/flagellin. Using CRISPR/Cas9 engineering of THP-1 cells to generate NLRC4- and/or NLRP3-deficient cells, we showed redundant roles for these proteins in inflammasome activation upon *S.* Typhimurium infection and that activation was predominantly dependent on the presence of flagellin. Dissecting the inflammasome pathways involved in ligand sensing, we found that *S.* Typhimurium flagellin activates NAIP/NLRC4 but, surprisingly, also NLRP3 through the canonical activation pathway in THP-1 cells. This was masked in hMDM in which multiple mechanisms are involved in the host response to *S.* Typhimurium. These findings suggest that flagellin can activate both the NLRP3 and NAIP/NLRC4 inflammasomes, potentially explaining some of the redundancy observed during *S.* Typhimurium infection in THP-1 cells. NLRP3 activation may therefore act as a “safety net” to preserve inflammasome activation during bacterial infection in the face of suboptimal NAIP/NLRC4 activation.

## Materials and Methods

### Human macrophage-like cells

#### THP-1 cells.

THP-1 cells were obtained from American Type Culture Collection. Cells were cultured in RPMI 1640 (42401-018; Life Technologies) supplemented with heat-inactivated 10% FCS (10100-147; Life Technologies), 2 mM GlutaMAX (35050-038; Life Technologies), and 1 mM sodium pyruvate (11360-039; Life Technologies) at 37°C and 5% CO_2_. Cells were split twice per week, and passage 10 was not exceeded. To differentiate THP-1 cells into macrophage-like cells, 5 × 10^4^ THP-1 cells/well were seeded into 96-well plates and treated with 200 ng/ml PMA (1544-5; BioVision) for 24 h. Medium was replaced with normal growth medium without PMA, and cells were rested for another 24 h prior to use in experiments.

#### Primary monocyte-derived macrophages.

Blood was obtained from healthy individuals from the in-house blood donation unit. The human biological samples were sourced ethically, and their research use was in accordance with the terms of the informed consents under an Institutional Review Board/Ethics Committee–approved protocol. PBMCs were isolated by density centrifugation using Histopaque-1077 Hybri-Max (H8889-500ML; Sigma-Aldrich). Isolated PBMCs were incubated with 100 μl CD14-binding MACS beads (130-050-201; Miltenyi Biotec) per 1 × 10^8^ cells for 15 min. Cells were magnetically sorted by positive selection using LS columns (130-042-401; Miltenyi Biotec). Isolated CD14^+^ cells were differentiated into macrophages using 100 ng/ml M-CSF (216-M-005/CF; R&D Systems) in RPMI 1640 medium supplemented with 5% heat-inactivated FCS and 2 mM GlutaMAX. Cells were seeded in 96-well plates at 1.8 × 10^5^ cells/well for ligand stimulations or at 1.5 × 10^5^ cells/well for bacterial infection or in 24-well plates at 1 × 10^6^ cells/well for RNA isolation and differentiated into hMDM for 5 d prior to use in experiments.

### Reagents and Abs

Custom-made recombinant *Salmonella* PrgI (P41784; UniProt) with an N-terminal 6xHis-TEV site (MHHHHHHENLYFQG) was expressed and purified from *Escherichia coli* by GenScript. Native ultrapure *S.* Typhimurium flagellin (tlrl-epstfla-5), ultrapure *Bacillus subtilis* flagellin (tlrl-pbsfla), the NLRP3 inhibitors MCC950 (inh-mcc), and glybenclamide (tlrl-gly) were purchased from InvivoGen. *E. coli* 0111:B4 LPS and nigericin were purchased from InvivoGen (LPS: tlrl-3pelps; nigericin: tlrl-nig) or Sigma (LPS: L5293-2ML; nigericin: SML1779-1ML). The caspase inhibitor z-VAD-fmk (FMK001) was purchased from R&D Systems. The cathepsin inhibitor Ca-047-Me was purchased from Enzo Life Sciences (BML-PI126-0001). The NADPH oxidase and NO synthetase inhibitor diphenyleneiodonium chloride (DPI) was purchased from Sigma (D2926-10MG). To detect proteins by Western blot analysis or microscopy, the following primary Abs were used: rabbit anti-human ASC (clone AL177; AdipoGen Life Sciences), rabbit anti-human NLRC4 (clone D5Y8E; Cell Signaling Technology), mouse anti-human NLRP3 (clone Cryo-2; AdipoGen Life Sciences), and mouse anti-human actin (clone AC-74; Sigma). No anti-NAIP Ab tested allowed detection of endogenous NAIP in human macrophages. Secondary Abs used for Western blot analysis were IRDye 680RD donkey anti-mouse IgG (926-68072; LI-COR Biosciences) and IRDye 800CW donkey anti-rabbit IgG (926-32213; LI-COR Biosciences). Secondary Abs used for microscopy were goat anti-rabbit IgG-Alexa Fluor (AF) 488 (A-11034; Invitrogen) or goat anti-rabbit IgG-AF546 (A-11035; Invitrogen).

### Genome editing

KO THP-1 cells were generated by CRISPR/Cas9 genome editing. Gene-specific CRISPR RNAs (crRNAs) were selected from the sgRNA Designer by the Broad Institute. Sequences of crRNAs are shown in Table I. Custom site-specific crRNA, *trans*-activating crRNA (tracrRNA) (1072534), and recombinant Cas9 protein (1081059) were ordered from Integrated DNA Technologies. Ribonucleoprotein complexes consisting of annealed crRNA and tracrRNA and Cas9 protein were assembled and cells electroporated using the Amaxa 2D or 4D electroporator (Lonza). Single crRNAs were used to target genes of interest or combinations of crRNA to target multiple genes at the same time. For Amaxa 2D electroporation, complexes were generated by annealing 600 pmol crRNA with 600 pmol tracrRNA at 95°C for 5 min and incubating cooled-down annealed RNA with 366 pmol Cas9 for 15 min at room temperature. For electroporation with 4D Amaxa electroporator, ribonucleoprotein complexes were generated using 100 pmol crRNA and 100 pmol tracrRNA incubated in 3 μl RNA duplex buffer (11-01-03-01; Integrated DNA Technologies) and 61 pmol Cas9. To gain sufficiently high editing efficiencies, 1 × 10^6^ THP-1 cells were electroporated in buffer P3 (V4XP-3032; Lonza) on three consecutive days using the Amaxa 2D electroporator. A single electroporation of 0.25 × 10^6^ cells was sufficient with the 4D Amaxa electroporator. Cells were rested for 48 h after the final electroporation and then seeded into 96-well plates at a concentration of 10 cells/ml to gain single clones. Genotyping was performed as described (21). In short, DNA was extracted followed by a locus-specific PCR and a barcoding PCR to allow sequencing of the amplicons using the Illumina MiSeq. Site-specific primers contained a barcoding and linker sequence at the 5′ end followed by a site-specific sequence. The following barcoding and linker sequences were used for forward and reverse primers, respectively: 5′-ACACTCTTTCCCTACACGACGctcttccgatct-3′ and 5′-TGACTGGAGTTCAGACGTGTGctcttccgatct-3′. Site-specific primer sequences are listed in Table I. Clones were selected and characterized phenotypically and, where possible, by Western blot analysis. Experiments were performed with pools of four validated KO clones.

### Quantitative RT-PCR analysis

PMA-differentiated THP-1 cells or hMDM were primed with 200 ng/ml LPS for 3 h or left untreated. Then, 1 × 10^6^ cells were stabilized in RNAprotect Cell Reagent (76527; QIAGEN) prior to further processing of samples. Total RNA was extracted using RNeasy Mini Kit (74106; QIAGEN) together with QIAshredder (79656; QIAGEN) columns. Isolated RNA was DNase treated using the TURBO DNA-free Kit (AM190; Thermo Fisher Scientific) and reverse transcribed into cDNA using the High-Capacity cDNA Reverse Transcription Kit (4366813; Thermo Fisher Scientific). Real-time quantitative PCR was performed on a Roche LightCycler 480 using the following TaqMan probes purchased from Thermo Fisher Scientific: NLRC4 (Hs00892666_m1), NAIP (Hs03037952_m1), NLRP3 (Hs00918082_m1), IL-1B (Hs01555410_m1), IL-18 (Hs01038788_m1), ACTB (Hs99999903_m1), RPL37A (Hs01102345_m1), and EIF2B2 (Hs00204540_m1). Crossing point (Cp) values were determined by the LightCycler software version SW1.5.1.62 using the Mono color hydrolysis probe program. Data analysis was performed as described in detail previously (22). Data are presented as fold change expression + SD over unstimulated THP-1 cells normalized for ACTB, RPL37A, and EIF2B2.

### Inflammasome stimulations

To trigger inflammasome activation, unprimed cells were transfected in OptiMem using equimolar amounts of the needle protein PrgI or *S.* Typhimurium flagellin by lipofection. For flagellin from *B. subtilis*, twice the molar amount was used. For this, 70 ng recombinant PrgI protein, 400 ng *S.* Typhimurium–derived flagellin, or 528 ng flagellin from *B. subtilis* were used together with 0.4 μl Lipofectamine 2000 (Invitrogen) per well in a 96-well format. Lipofectamine–protein complexes were formed in OptiMem medium and added to cells for stimulations for 4 or 24 h. Noncanonical NLRP3 activation was initiated by transfecting 100 ng LPS into cells using 0.4 μl lipofectamine. Lipofectamine alone was used as a control. To activate the canonical NLRP3 inflammasome, cells were primed with 200 ng/ml LPS for 3 h, and following a media change, NLRP3 was activated with 5 μM nigericin or cells were treated with DMSO or ethanol (EtOH) as a vehicle control for 1 or 21 h. Cells were pretreated for 1 h prior to stimulation with 1 μM MCC950 or 25 μg/ml glybenclamide to inhibit NLRP3 activation, 10 μM DPI to inhibit cellular reactive oxygen species (ROS) production, and 100 μM cathepsin inhibitor Ca-047-Me or indicated amounts of the pan-caspase inhibitor z-VAD-fmk.

### Salmonella infection

THP-1 cells (5 × 10^4^ cells/well) or hMDM (1.5 × 10^5^ cells/well) were infected at the indicated multiplicity of infection (MOI) with the wild-type *S.* Typhimurium SL1344 strain, with the flagellin-deficient mutant strain *S.* Typhimurium Δ*fljB*Δ*fliC*, or with the T3SS/flagellin-deficient strain Δ*prgJ*Δ*fljB*Δ*fliC*. The mutant strains, infection conditions, and readouts used in these experiments have been described previously (5).

### ASC speck analysis

Imaging of ASC speck formation has been described previously (23). In short, THP-1 cells were fixed poststimulation in 4% paraformaldehyde (in PBS) for 15 min. Fixation buffer was removed, and cells were then permeabilized and blocked in 50 μl PBS containing 3% FCS, 0.5% BSA, and 0.5% saponin for 15 min. Rabbit anti-human ASC Ab (clone AL177, 1:500; AdipoGen Life Sciences) was diluted in permeabilization/blocking buffer, and 50 μl was added to the wells, thereby diluting the Ab solution further to reach the final concentration. Cells were incubated with Ab solution overnight at 4°C. Ab solution was removed, and cells were carefully washed twice with 100 μl PBS. Cells were incubated with 1 μg/ml goat anti-rabbit AF488 and 0.5–1 μg/ml CellMask Blue for 1 h at room temperature. Cells were washed once in PBS. Cells were submerged in PBS, and six images per well were automatically acquired on the DAPI and 488 channels of an INCell 2200 HCS imager. Analysis and quantification of the images was performed in Columbus version 2.8.3 (PerkinElmer). The analysis script was built to filter images; identify nuclei and count cells; remove big/small cell clumps from the analysis; identify ASC specks based on fluorescence intensity, size, and fluorescence intensity over size; and identify specks inside nonpyroptotic cells. Cells typically formed a single speck per cell. The total cell count was calculated by the addition of the number of cells identified and number of specks found. Typically, 2000–3000 cells/well were analyzed. The percentage of specking cells was calculated by dividing the number of specks by total cell count and multiplied by 100.

### ELISA analysis

To determine IL-1β and IL-18 cytokine levels in culture supernatants, ELISAs were performed using the human IL-1β/IL-1F2 (DY201) and total human IL-18 (DY318-05) DuoSet ELISA kits from R&D Systems. ELISA assays were performed according to manufacturer instructions with the exception that assays were downscaled using 50 and 25 μl coating Ab solution in 96-well plates and 96-well half-area plates, respectively. All other reagents were downscaled accordingly. To determine IL-1β levels, supernatants were diluted as appropriate, whereas neat supernatants were used for IL-18 ELISAs. Plates were read on a SpectraMax i3 at 450 and 540 nm for background measurement, which were subtracted. Cytokine concentration was determined by extrapolation from the standard curve.

### Lactate dehydrogenase cytotoxicity assay

To determine cell viability, the lactate dehydrogenase (LDH) assay kit (88954) from Pierce Biotechnology was used. The remaining cell viability was determined by lysing cells in 50 μl 1× lysis buffer, and 50 μl LDH substrate was added and incubated until a sufficient colorimetric change was visible. The reaction was stopped using the LDH stop solution, and the plate was read at 490 and 680 nm for background measurement, which were subtracted. Untreated sample was set at 100% viability, and the proportion of remaining cell viability was determined for all other conditions.

### Western blotting

To assess the loss of protein expression in KO cells, PMA-differentiated THP-1 cells were lysed in RIPA lysis buffer (89900; Pierce Biotechnology) containing Halt Protease and phosphatase inhibitor mixture (78441; Thermo Fisher Scientific). Protein concentration was determined using a BCA Assay Kit (23227; Pierce Biotechnology). Equal amount of protein was loaded onto NuPage 4–12% Bis-Tris gels (NP0335BOX; Invitrogen) and separated by SDS-PAGE using MOPS buffer (NP0001; Invitrogen). Gels were blotted at 30 V for 2 h onto Immobilon-FL PVDF membranes (IPFL00010, 0.45 μm pore size; MilliporeSigma) by wet transfer using transfer buffer (NP0006-1; Invitrogen). Membranes were blocked in LI-COR Odyssey TBS blocking solution (927-50000; LI-COR Biosciences) for 30 min. Membranes were incubated with primary Abs overnight at 4°C or for 2 h at room temperature. Membranes were washed in TBS containing 0.5% Tween 20 and incubated in secondary Abs for 1 h at room temperature. Blots were washed in TBS containing 0.5% Tween 20 and imaged using an LI-COR Odyssey CLx Imager.

### Statistical analysis

Statistical analysis was performed in Prism GraphPad 5.0.4. Statistical significance was determined using one-way ANOVA followed by a Bonferroni multiple comparison test for ligand experiments in wild-type cells. For ligand stimulation in wild-type versus KO cell lines, THP-1 versus hMDM or in the presence of inhibitors, or for *S.* Typhimurium infection experiments, statistical analysis was performed using a two-way ANOVA followed by a Bonferroni posttest. The *p* values <0.05 were considered significant.

## Results

### NLRP3 and NLRC4 are both required for *S.* Typhimurium–induced inflammasome activation in THP-1 cells

**FIGURE 1.**
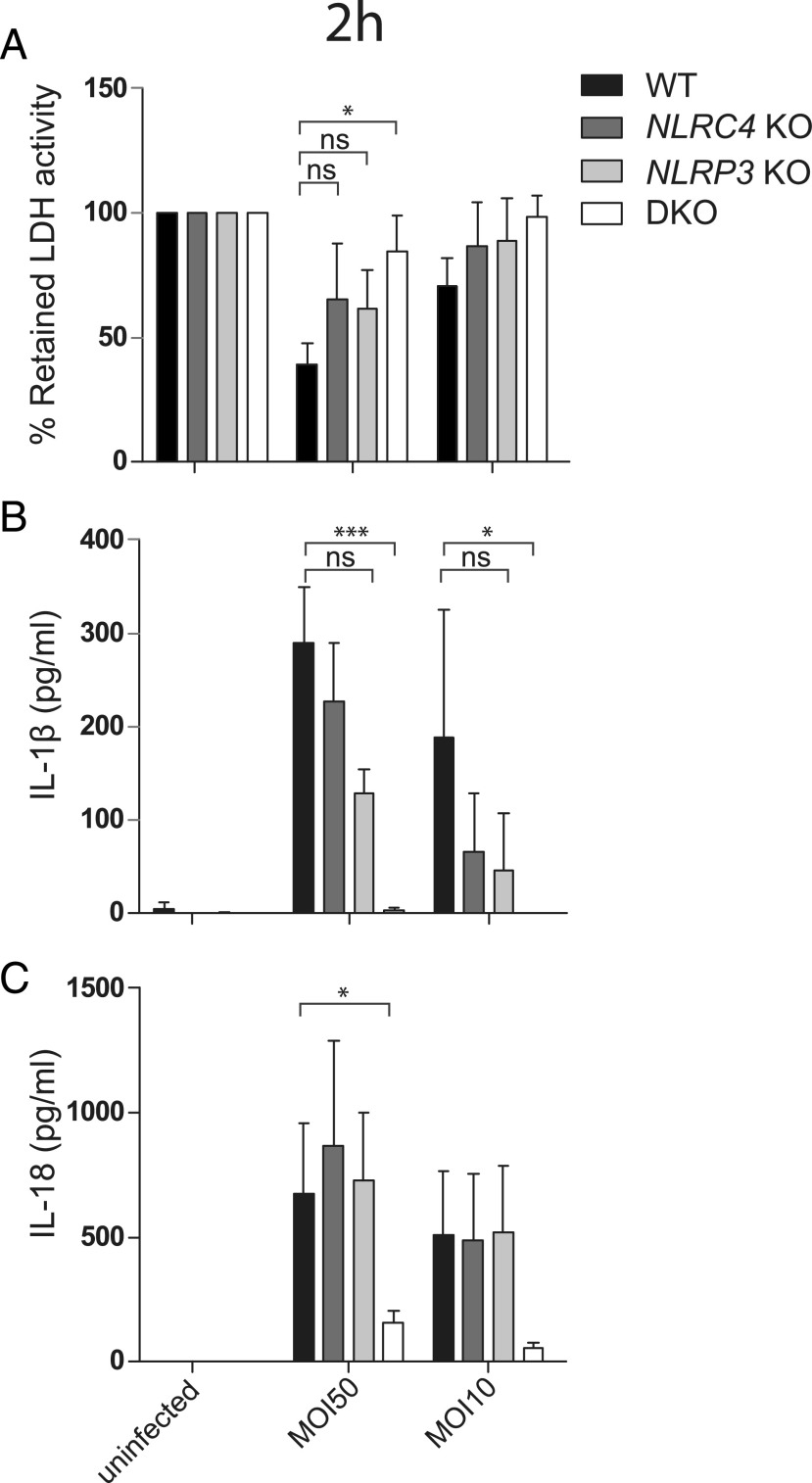
Inflammasome activation in THP-1 cells requires both NLRP3 and NLRC4 in response to *Salmonella* infection. THP-1 wild-type, *NLRC4* KO, *NLRP3* KO, and *NLRC4/NLRP3* DKO cells were infected with *S.* Typhimurium at MOI 50 or 10. Cell viability (**A**) and cytokine secretion (**B** and **C**) were assessed at 2 h postinfection. Mean with SD of two (IL-1β) or three (cell viability, IL-18) independent experiments (three replicates/experiment) are shown. Statistical analysis was performed using two-way ANOVA followed by a Bonferroni posttest; *p < 0.05, ***p < 0.001.

In human and murine macrophages, NAIP/NLRC4 and NLRP3 are triggered to form inflammasomes upon infection with *S.* Typhimurium (4–7). We set out to characterize the contribution of these pathways in our human macrophage models in response to *S.* Typhimurium infection. To this end, we used CRIPSR/Cas9 technology to generate KO THP-1 cells lacking NLRC4, NLRP3, or both NLRC4 and NLRP3 (double KO [DKO]) (Table I). Monoclonal KO cells were generated, genotyped by MiSeq analysis, and characterized by Western blot analysis (Supplemental Fig. 1A–F). Four individually validated THP-1 KO clones deficient for NLRC4, NLRP3, or both NLRC4 and NLRP3 were pooled for experiments and compared with parental unedited cells. Following *S.* Typhimurium infection of PMA-differentiated wild-type THP-1 cells or receptor-deficient KO cells, IL-1β and IL-18 release and cell viability by residual LDH activity were determined at 2, 6, or 24 h. At 2 h postinfection, cell death was markedly reduced in the DKO (16%) in comparison with wild-type cells (61%) at MOI 50 (Fig. 1A). These DKO cells failed to secrete IL-1β or IL-18 in response to *S.* Typhimurium, but KO of either *NLRP3* or *NLRC4* alone was not sufficient to significantly decrease cytokine release (Fig. 1B, 1C). At later time points, IL-1β production was minimal in the *NLRP3* KO and the DKO cells (Supplemental Fig. 2A–C). Together, these data suggest that both NLRP3 and NLRC4 contribute to the inflammasome response to infection with *S.* Typhimurium of human macrophages.

**Table I.** Sequences of crRNA used to target indicated genes and site-specific primers used to amplify amplicons for genotyping

Target	crRNA Sequence	Sequence Primer Forward, 5′-3′	Sequence Primer Reverse, 5′-3′	Clones
*NLRC4*	5′-GACGTCTCATGAGCCAGAGG-3′	5′-CTAGCTCTGGAGGGTGTGTTCT-3′	5′-AATGGAAACCATTTTCTGCAAG-3′	1, 2
*NLRC4*	5′-GGACCAACACCATCACCGCG-3′	5′-CCATACCCCATCTTTTCTGAAC-3′	5′-GAATTTGAACTTGGTCAGAGCC-3′	3, 4
*NLRP3*	5′-AAGGAAGAAGACGTACACCG-3′	5′-TCAGTCTGATTCAGGAGAACGA-3′	5′-CTCCTCAAACAGGATTTTCTGG-3′	1–4
*NLRC4/NLRP3*	5′-GACGTCTCATGAGCCAGAGG-3′ (NLRC4) 5′-AAGGAAGAAGACGTACACCG-3′ (NLRP3)	5′-CTAGCTCTGGAGGGTGTGTTCT-3′ 5′-TCAGTCTGATTCAGGAGAACGA-3′	5′-AATGGAAACCATTTTCTGCAAG-3′ 5′-CTCCTCAAACAGGATTTTCTGG-3′	1–4
*NAIP*	5′-GACTTGCGTCCTTCAGGAAC-3′	5′-AGACTCCCCATAGAAGACCACA-3′	5′-CTGTAAAGACAAAGCCAGCCTC-3′	1–4

All site-specific primers contained a barcode and linker sequence at the 5′ terminus (see text for details), which is not shown.

### Flagellin contributes to inflammasome activation during *S.* Typhimurium infection in THP-1 cells

**FIGURE 2.**
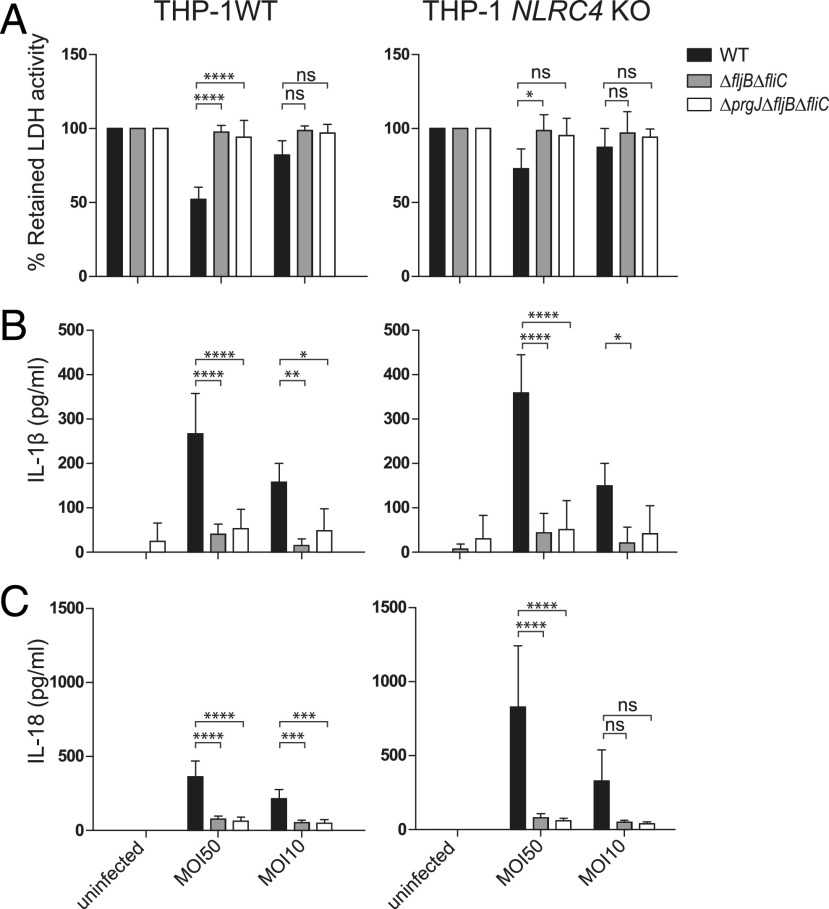
Flagellin-deficient *S.* Typhimurium is defective in activating the inflammasome in infected THP-1 cells. THP-1 wild-type and *NLRC4* KO cells were infected with *S.* Typhimurium at MOI 50 or 10. Cell viability (**A**) and cytokine secretion (**B** and **C**) were assessed at 2 h postinfection. Mean with SD of three independent experiments (three replicates/experiment) are shown. Statistical analysis was performed using two-way ANOVA followed by a Bonferroni posttest. *p < 0.05, **p < 0.01, ***p < 0.001, ****p < 0.0001.

Our data confirm that both NLRP3 and NAIP/NLRC4 contribute to the inflammasome response in human macrophages upon infection with *S.* Typhimurium, but the relative contribution of various ligands of this bacterium is unclear. Flagellin and T3SS needle and rod proteins from *S.* Typhimurium activate the murine and human NAIP/NLRC4 inflammasome (24). To assess their role in *S.* Typhimurium–induced inflammasome activation, we used flagellin- and T3SS/flagellin-deficient *S.* Typhimurium strains to infect parental THP-1 and *NLRC4* KO cells. *S.* Typhimurium lacking *fljB* and *fliC* do not produce flagellin, and bacteria lacking *prgJ* are unable to form a T3SS, which prevents delivery of proteins such as PrgI, but also flagellin, into the cytosol (25). At 2 h postinfection, wild-type THP-1, and to a lesser extent *NLRC4* KO cells, infected with wild-type *S.* Typhimurium at MOI 50 showed a clear decrease in cell viability, whereas cells infected with the flagellin-deficient strain Δ*fljB*Δ*fliC* and the T3SS/flagellin-deficient strain Δ*prgJ*Δ*fljB*Δ*fliC* did not induce cell death (Fig. 2A). The viability of wild-type *S.* Typhimurium–infected cells further decreased over the next 22 h, but cells infected with strains lacking flagellin or T3SS/flagellin remained mostly viable (Supplemental Fig. 2D). At 2 h postinfection, IL-1β and IL-18 production was reduced in both parental and *NLRC4* KO THP-1 cells infected with the flagellin- or T3SS/flagellin-deficient strain in comparison with infection with wild-type *S.* Typhimurium (Fig. 2B, 2C). At 24 h postinfection, however, the cells infected with the strains lacking flagellin and T3SS/flagellin secreted cytokines (Supplemental Fig. 2E, 2F). This observation may be explained by the rapid cell death induced by wild-type *S.* Typhimurium reducing the number of residual cells available for cytokine processing and delayed cell death induced by the other strains (Fig. 2A). Taken together, these data suggest that flagellin is important for cytokine production and cell death during *S.* Typhimurium infection in THP-1 cells without further enhancement by the T3SS system.

### Recombinant *S.* Typhimurium PrgI and native *S.* Typhimurium flagellin trigger inflammasome activation in human macrophages

**FIGURE 3.**
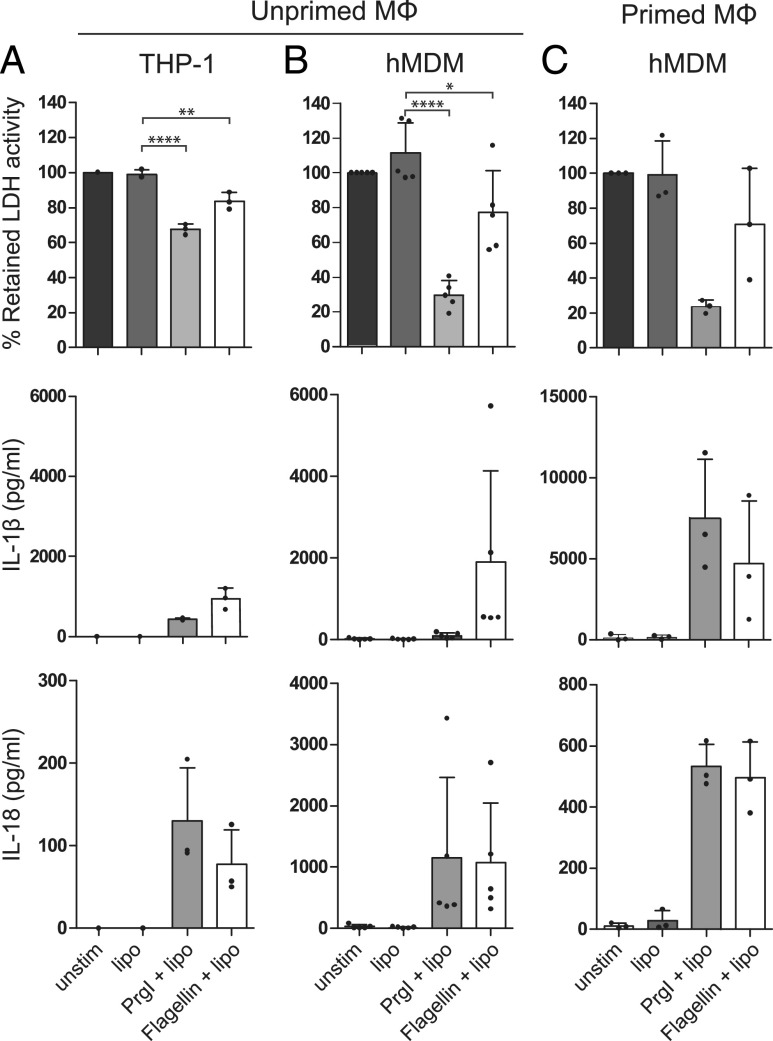
*Salmonella* ligands trigger inflammasomes in human macrophages. Unprimed THP-1 cells (**A**), hMDM (**B**), and LPS-primed hMDM (**C**) were transfected with recombinant needle protein PrgI or native *S.* Typhimurium flagellin using Lipofectamine 2000. Cells were assayed at 4 h for cell viability (A–C, top panel), IL-1β (A–C, middle panel), and IL-18 (A–C, bottom panel). Bars show mean of cell viability or cytokine secretion, and dots indicate replicate mean of individual experiments (THP-1, *n* = 3; three replicates/experiment) or donors (hMDM, *n* = 5 [unprimed], *n* = 3 [primed]; three replicates per donor). Statistical significance was determined using one-way ANOVA followed by a Bonferroni multiple comparison test. *p < 0.05, **p < 0.01, ****p < 0.0001.

To further characterize the response of THP-1 cells to *S.* Typhimurium infection, we investigated the inflammasome response induced by individual ligands in THP-1 cells and compared it with the responses in hMDM. It has been suggested that *S.* Typhimurium flagellin induces cell death only in primary hMDM but not in macrophage-like cell lines using an anthrax toxin delivery method (20). Cytosolic delivery of the T3SS needle protein PrgI from *S.* Typhimurium triggers the NAIP/NLRC4 inflammasome in the macrophage-like cell lines THP-1 and U937 (10). Native flagellin or recombinant needle protein PrgI from *S.* Typhimurium were delivered into unprimed human macrophages by lipofection. At 4 h poststimulation, PrgI transfection resulted in reduced cell viability in comparison with the transfection reagent control (lipo) or unstimulated THP-1 cells (Fig. 3A; top panel) and hMDM (Fig. 3B; top panel). In hMDM, a mean decrease in cell viability of 70% was measured for PrgI-transfected cells, whereas THP-1 cells showed a mean viability reduction of 30% (Fig. 3A, 3B; top panels). Flagellin had a less pronounced effect on cell viability than PrgI with a mean decrease of 23 and 17% at 4 h poststimulation in hMDM and THP-1 cells, respectively (Fig. 3A, 3B; top panels).

THP-1 cells released IL-1β in response to both *S.* Typhimurium proteins at 4 h poststimulation (Fig. 3A; middle panel). Unprimed hMDM released only low levels of IL-1β in response to PrgI, despite undergoing substantial cell death (Fig. 3B; middle panel). Flagellin induced less cell death than PrgI, but it resulted in higher levels of released IL-1β in both macrophage models (Fig. 3A, 3B; middle panels). We hypothesized that this disconnect between cell death and IL-1β release for PrgI, but not flagellin, was probably due to cell priming by extracellular flagellin. Flagellin is the ligand of TLR5, which is expressed on the cell surface by a number of innate immune cells, including macrophages, leading to NF-κB–mediated proinflammatory cytokine expression (26–29). Pro–IL-18 is also processed by caspase-1 and secreted in an inflammasome-dependent manner but is constitutively transcribed and translated. In THP-1 cells and hMDM, similar levels of IL-18 were secreted upon stimulation with PrgI or flagellin, suggesting that inflammasomes are triggered by both stimuli and that pro–IL-1β expression is upregulated presumably through activation of TLR5 by extracellular flagellin but not PrgI in hMDM (Fig. 3A, 3B; bottom panels). To confirm that PrgI transfection can result in efficient IL-1β secretion in hMDM, we measured IL-1β release from LPS-primed hMDM in response to PrgI or flagellin (Fig. 3C; middle panel). Primed cells released high levels of IL-1β and IL-18 in response to both stimuli, whereas cell viability was similar to unprimed hMDM (Fig. 3C, middle and bottom panel). This demonstrates that full inflammasome activation can be triggered by PrgI in hMDM. Together, these data show that both human macrophage models responded to the *S.* Typhimurium ligands PrgI and flagellin, leading to a reproducible inflammasome activation.

### Flagellin activates the inflammasome in a NAIP/NLRC4- and NLRP3-dependent manner in THP-1 cells

**FIGURE 4.**
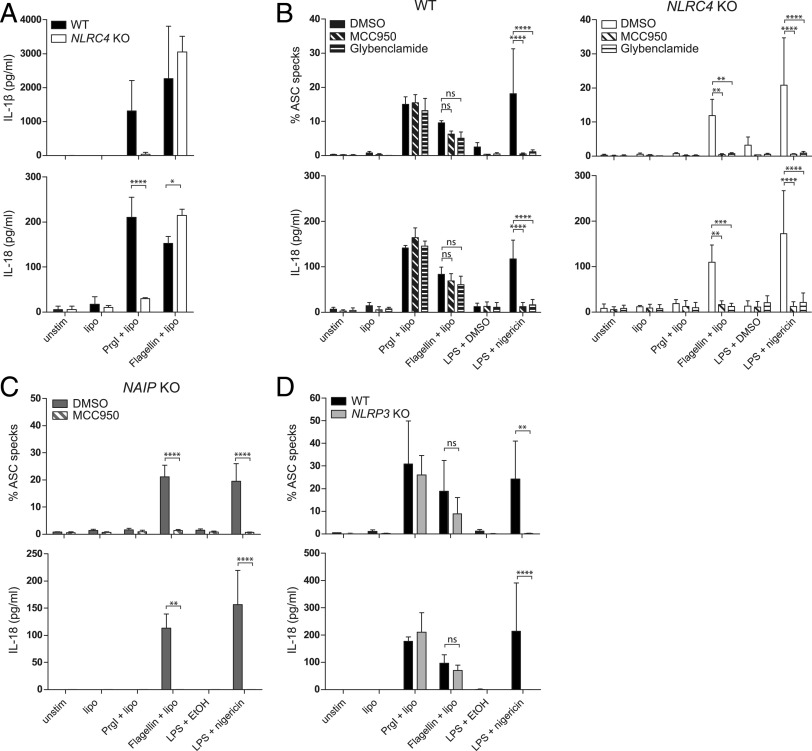
Recombinant PrgI triggers the NAIP/NLRC4 inflammasome, whereas flagellin activates the NAIP/NLRC4 and the NLRP3 inflammasome in THP-1 cells. Parental wild-type THP-1 (**A**, **B**, and **D**), *NLRC4* KO (A and B), *NAIP* KO (**C**), or *NLRP3* KO (D) cells were transfected with PrgI or flagellin (A–D) or stimulated with LPS+ nigericin (B–D) in the presence of NLRP3 inhibitors MCC950 (B and C) or glybenclamide (B). ASC speck formation (B–D) and IL-1β (A) and IL-18 (A–D) release were determined at 4 h poststimulation. Mean with SD of at least three individual experiments (two replicates/experiment) is displayed. Statistical analysis was performed using two-way ANOVA followed by a Bonferroni posttest. *p < 0.05, **p < 0.01, ***p < 0.001, ****p < 0.0001.

PrgI and flagellin are well-characterized activators of the Naip1/NLRC4 and Naip5/6/NLRC4 inflammasome in murine macrophages, respectively (30). Knockdown of NAIP in hMDM has been reported to lead to reduced inflammasome activation in response to flagellin and PrgJ (18). Using THP-1 *NLRC4* KO cells, we assessed whether PrgI and flagellin mediated inflammasome activation, as expected, through the NAIP/NLRC4 axis. Transfection of THP-1 *NLRC4* KO cells with the *S.* Typhimurium ligand PrgI failed to induce the release of IL-1β or IL-18, confirming that PrgI triggers the NAIP/NLRC4 inflammasome in THP-1 cells (Fig. 4A). *NLRC4* KO cells, unexpectedly, responded to flagellin transfection with IL-1β and IL-18 levels similar to those produced by wild-type cells, suggesting that NLRC4 is either not required or is only partially responsible for flagellin-mediated inflammasome activation in THP-1 cells (Fig. 4A).

NLRC4 colocalizes or coimmunoprecipitates with NLRP3 during *S.* Typhimurium infection of murine and human macrophages (5, 7), and we observed that inflammasome activation was dependent on both NLRC4 and NLRP3 in *S.* Typhimurium–infected THP-1 cells (Fig. 1A–C). To determine the contribution of NLRP3 to flagellin-mediated inflammasome activation, we incubated *NLRC4* KO cells with the NLRP3 inhibitors glybenclamide or MCC950 prior to stimulation. To measure inflammasome activation in a more direct manner than through cytokine release or cell death, we also imaged endogenous ASC speck formation, allowing robust quantification of the NLR-driven inflammasome response (23). In the presence of the NLRP3 inhibitors, ASC speck formation and cytokine release, as expected, were markedly reduced at 4 h in nigericin-stimulated but not PrgI-transfected cells, confirming the selectivity of these inhibitors for NLRP3 (Fig. 4B). Flagellin-mediated inflammasome activation in *NLRC4* KO cells was lost upon treatment with glybenclamide or MCC950, showing that in these cells, flagellin activated NLRP3 (Fig. 4B, right panels). A modest decrease in ASC speck formation and cytokine release was observed in flagellin-stimulated wild-type cells pretreated with MCC950 or glybenclamide suggesting that in THP-1 wild-type cells, inflammasome activation was not solely dependent on NLRP3 (Fig. 4B, left panels; Supplemental Fig. 3A, left panel). Taken together, these data suggest that both NLRP3 and NLRC4 are activated by flagellin in THP-1 wild-type cells.

NLRC4 cannot sense flagellin or PrgI directly; ligand binding and inflammasome activation is mediated by NAIP (10, 14). *NAIP* KO cells (Supplemental Fig. 1G) failed to respond to PrgI with ASC speck formation or cytokine release, demonstrating the inactivity of the NAIP/NLRC4 pathway in these cells (Fig. 4C, Supplemental Fig. 3B). To determine whether flagellin-mediated NLRP3 activation also requires NAIP, THP-1 *NAIP* KO cells were stimulated with flagellin in the presence of the NLRP3 inhibitor MCC950. Flagellin triggered an inflammasome response in *NAIP* KO cells, but this was fully inhibited by MCC950, demonstrating that the inflammasome response to flagellin was NLRP3 dependent but NAIP independent in the absence of the canonical flagellin detection system (Fig. 4C, Supplemental Fig. 3B).

To validate that the NAIP/NLRC4 activation was independent of NLRP3 in THP-1 cells, *NLRP3* KO cells were stimulated with PrgI, flagellin, or nigericin. The NAIP/NLRC4 activator PrgI, but not the canonical NLRP3 activator nigericin, triggered an inflammasome response in *NLRP3* KO cells, validating these KO cells phenotypically. Flagellin, like PrgI, triggered an inflammasome response in *NLRP3* KO cells (Fig. 4D, Supplemental Fig. 3C), confirming that flagellin could trigger inflammasome responses independently of NLRP3. Together, these data suggest that native *S.* Typhimurium flagellin can directly activate NAIP/NLRC4 and the NLRP3 inflammasome in human THP-1 cells.

### Flagellin triggers the canonical NLRP3 response in THP-1 cells through ROS production and/or cathepsin activity

**FIGURE 5.**
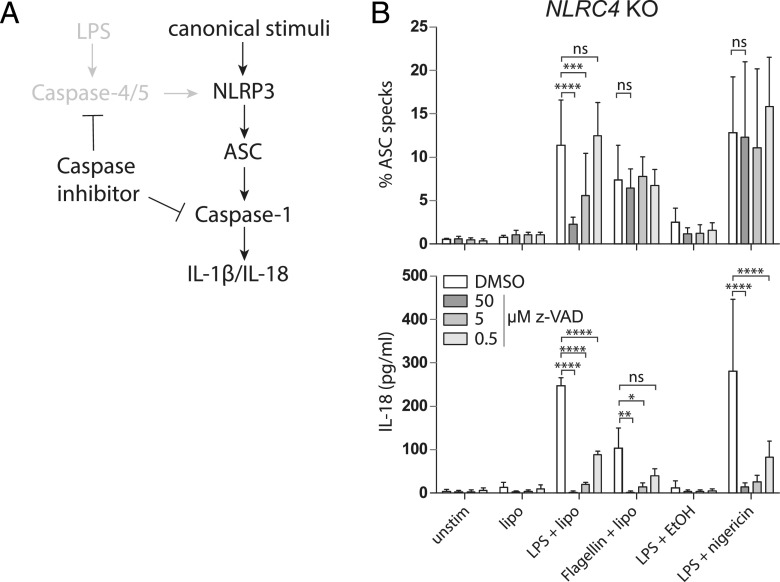
Flagellin activates the canonical NLRP3 inflammasome in THP-1 cells. (**A**) Schematic figure of canonical NLRP3 activation is shown in black; noncanoncial caspase-4/-5–mediated pathway is shown in gray. (**B**) THP-1 *NLRC4* KO cells were transfected with flagellin or LPS or stimulated with extracellular LPS and EtOH or nigericin in the presence of the pan-caspase inhibitor z-VAD-fmk. ASC speck formation and IL-18 release were determined at 4 h poststimulation. Mean with SD of at least three individual experiments (two replicates/experiment) is displayed. Statistical analysis was performed using two-way ANOVA followed by a Bonferroni posttest. *p < 0.05, **p < 0.01, ***p < 0.001, ****p < 0.0001.

LPS is a constituent of the cell wall of Gram-negative bacteria, such as *Salmonella*. Cytoplasmic LPS triggers noncanonical NLRP3 activation in a caspase-4/-5–dependent manner (31). Our studies employed ultrapure flagellin, but to exclude the possibility any potential contaminating LPS was responsible for noncanonical NLRP3 inflammasome activation, we sought to distinguish canonical and noncanonical inflammasome activation in this system. We reasoned that we could discriminate between canonical and noncanonical inflammasome activation by assessing ASC speck formation in the presence of a pan-caspase inhibitor. This inhibitor should selectively prevent caspase-4/5–dependent NLRP3 activation and ASC speck formation but not canonical NLRP3 activation (Fig. 5A). ASC speck formation was unaffected in the presence of the pan-caspase inhibitor z-VAD-fmk when stimulated with nigericin, but high concentrations of the caspase inhibitor decreased ASC speck formation upon cytosolic transfection of LPS (Fig. 5B, top panel). In contrast, z-VAD-fmk decreased cytokine secretion in a concentration-dependent manner for both stimuli (Fig. 5B, bottom panel).

We then asked whether *Salmonella* flagellin activated the NLRP3 inflammasome through the canonical or noncanonical pathway. Flagellin was transfected into *NLRC4* KO cells in the presence of z-VAD-fmk, which diminished cytokine secretion, as expected, because of the inhibition of caspase-1–mediated cytokine processing. ASC speck formation, however, was not altered, similar to what was seen in response to stimulation with the canonical NLRP3 activator nigericin (Fig. 5B). This demonstrates that the NLRP3 activation induced by *Salmonella* flagellin in THP-1 cells triggered the canonical and not the noncanonical NLRP3 pathway (Fig. 5A, 5B).

**FIGURE 6.**
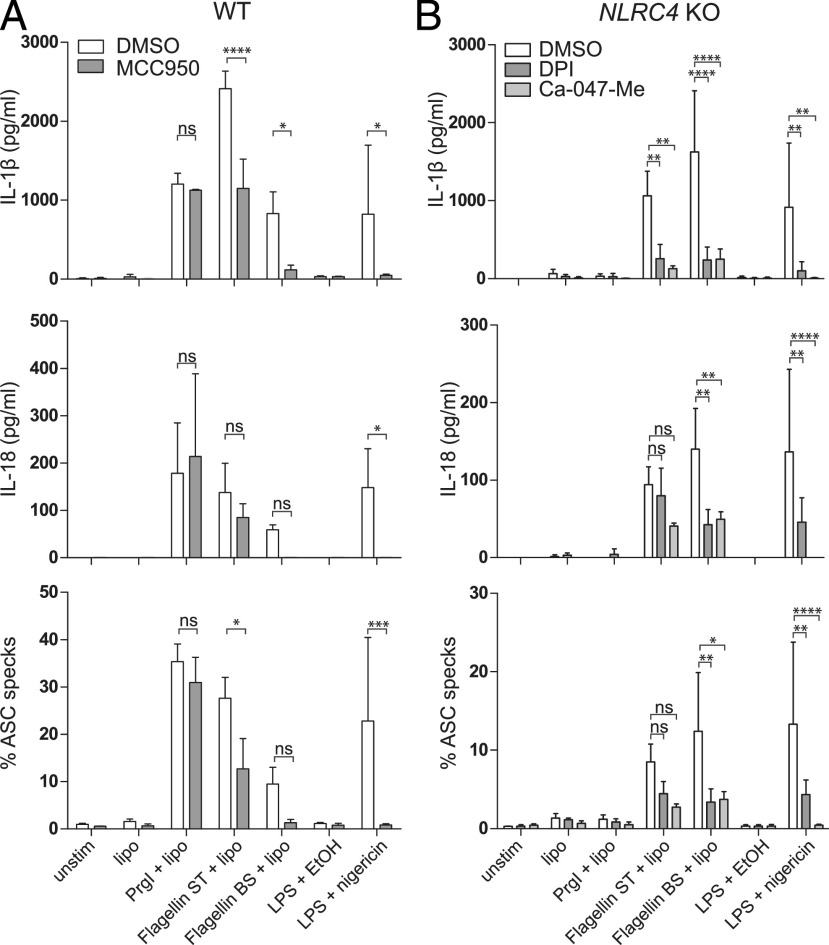
Flagellin-mediated NLRP3 inflammasome is dependent on ROS production and cathepsin activity. (**A**) THP-1 cells were transfected with equimolar amounts of PrgI or *S.* Typhimurium flagellin (ST) or twice the molar amount of *B. subtilis* flagellin (BS) or stimulated with LPS and EtOH or nigericin in the presence of the NLRP3 inhibitor MCC950. IL-1β and IL-18 release as well as ASC speck formation were determined at 4 h poststimulation. (**B**) THP-1 *NLRC4* KO cells were transfected with PrgI, *Salmonella*-, or *B. subtilis*–derived flagellin or stimulated with extracellular LPS and EtOH or nigericin in the presence of the ROS inhibitor DPI or the cathepsin inhibitor Ca-047-Me. ASC speck formation and cytokine release were determined at 4 h poststimulation. Mean with SD of three individual experiments (two replicates/experiment) is displayed. Statistical analysis was performed using two-way ANOVA followed by a Bonferroni posttest. *p < 0.05, **p < 0.01, ****p < 0.0001.

Gram-positive bacteria do not synthesize LPS, so their flagellin will not be contaminated with this bacterial ligand. To provide further evidence to support our finding that flagellin triggers canonical NLRP3 activation, we transfected native flagellin derived from the Gram-positive bacterium *B. subtilis* into wild-type THP-1 cells. *B. subtilis* flagellin triggered lower levels of inflammasome activity than *Salmonella* flagellin in this human macrophage model, so we doubled the amount of protein transfected to increase stimulation and aid the characterization of the inflammasomes triggered by this protein. The NLRP3 inhibitor MCC950 inhibited ASC speck formation and cytokine secretion in response to *B. subtilis* flagellin (Fig. 6A); however, it failed to trigger the NAIP/NLRC4 inflammasome. This experiment suggests that *B. subtilis* flagellin can also trigger the NLRP3 inflammasome in THP-1 cells, supporting the concept that bacterial flagellins can trigger canonical NLRP3 activation in human macrophages.

Several pathways are implicated in NLRP3 activation, including K^+^ efflux, ROS production, cathepsin release, or dispersion of the *trans*-Golgi network (32). To determine the mechanism by which flagellin activated the NLRP3 inflammasome, we used the pharmacological inhibitors DPI and Ca-047-Me, which block cellular ROS production and cathepsin activity, respectively. Nigericin-mediated ASC speck formation and cytokine secretion was reduced with either inhibitor, showing that ROS production and/or cathepsin activity can activate NLRP3 (Fig. 6B). Inhibitor-treated *NLRC4* KO cells displayed reduced IL-1β secretion and ASC speck formation in response to both *Salmonella*- and *B. subtilis*–derived flagellin (Fig. 6B), indicating that bacterial flagellins activate both of these pathways to trigger NLRP3 activation.

### NAIP mRNA expression is elevated in hMDM in comparison with THP-1 cells

**FIGURE 7.**
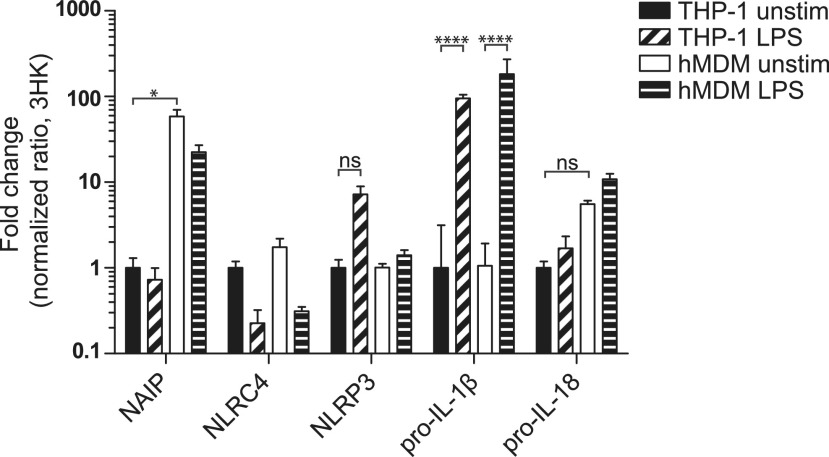
THP-1 cells and hMDM express similar levels of NLRC4 and NLRP3, but NAIP mRNA levels are higher in hMDM. THP-1 cells and hMDM were treated with LPS for 3 h or left unstimulated. Total RNA was isolated, cDNA generated, and a quantitative RT-PCR performed to assess levels of NAIP, NLRC4, NLRP3, pro–IL-1β, and pro–IL-18. Levels were normalized against three housekeeping genes (β-actin, RPL37A, and EIF2B2) and are shown as normalized ratio to THP-1–unstimulated cells. Mean with SD of three individual experiments (THP-1: three replicates/experiment; hMDM: two donors in triplicate/experiment) is displayed. Statistical analysis was performed using two-way ANOVA followed by a Bonferroni posttest. *p < 0.05, ****p < 0.0001.

Our data collectively support a hypothesis whereby flagellin may be the link that activates NLRP3 as well as NAIP/NLRC4 during *S.* Typhimurium infection of macrophages. The efficiency of NAIP/NLRC4 activity in THP-1 cells, particularly in response to flagellin, is thought to be limited because THP-1 cells express lower levels of NAIP and/or a specific isoform of NAIP mRNA compared with hMDM cells (20). Comparing mRNA levels of NAIP, NLRC4, NLRP3, pro–IL-1β, and pro–IL-18 under unprimed and LPS-primed conditions in hMDM and THP-1, we found that THP-1 cells express similar mRNA levels of NLRC4, NLRP3, and pro–IL-1β under resting conditions as hMDM (Fig. 7A). NAIP and pro–IL-18 mRNA were 58-fold and 5-fold higher expressed in hMDM than in THP-1 cells, respectively, confirming earlier observations on NAIP expression levels (20) (Fig. 7A). As expected, pro–IL-1β levels increased significantly upon LPS stimulation in THP-1 cells and hMDM by 95-fold and 180-fold, respectively. Only in THP-1 cells, a clear, but insignificant increase in NLRP3 mRNA levels was observed upon LPS treatment. Interestingly, NAIP and NLRC4 mRNA levels were reduced upon LPS stimulation in hMDM and THP-1 cells. This trend was more pronounced in hMDM than THP-1 cells, but statistically NS in both macrophage models. This may point toward an LPS-driven regulatory mechanism of NAIP/NLRC4, although it remains unclear how protein levels and their functionality are affected in this time frame.

### The T3SS is sufficient to induce inflammasome activation upon *S.* Typhimurium infection of hMDM

**FIGURE 8.**
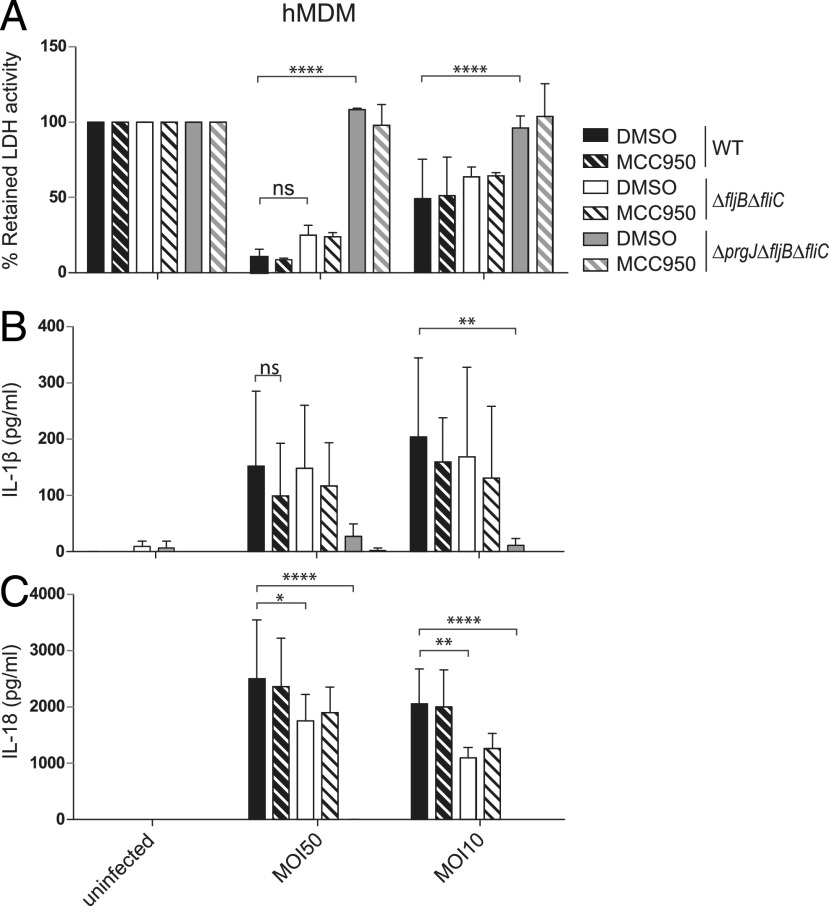
In hMDM, flagellin-deficient *S.* Typhimurium is sufficient to trigger inflammasome activation, which is NLRP3 independent. hMDM were infected with *S.* Typhimurium at MOI 50 or 10. Cell viability (**A**) and cytokine secretion (**B** and **C**) were assessed at 2 h postinfection. Mean with SD of three different donors (three replicates per donor) is displayed. Statistical analysis was performed using two-way ANOVA followed by a Bonferroni posttest. *p < 0.05, **p < 0.01, ****p < 0.0001.

In our characterization of the flagellin-mediated inflammasome activation in THP-1 cells, we showed that this ligand could activate NLRP3 in the absence of NAIP or NLRC4. Primary hMDM have much higher levels of NAIP in comparison with THP-1 cells, and our investigatory tools in hMDM are restricted to NLRP3 inhibitors and bacterial strains deficient for some of the inflammasome-activating ligands, thus it is challenging to determine whether flagellin also triggers the canonical NLRP3 pathway in hMDM. Flagellin contributed to inflammasome activation in *S.* Typhimurium–infected THP-1 cells, so we determined whether this was also the case in primary hMDM by infecting these cells with the different *S.* Typhimurium strains. The wild-type *S.* Typhimurium strain, but not the T3SS/flagellin-deficient Δ*prgJ*Δ*fljB*Δ*fliC* strain, induced 90% cell death at 2 h postinfection at MOI 50 (Fig. 8A). The flagellin-deficient strain Δ*fljB*Δ*fliC*, however, induced 75% cell death at 2 h postinfection at MOI 50 in hMDM (Fig. 8A). This suggests that the needle and rod proteins delivered by the T3SS were sufficient to induce an inflammasome response in hMDM. Cells infected with wild-type or the flagellin-deficient *S.* Typhimurium strain Δ*fljB*Δ*fliC* secreted comparable amounts of IL-1β and IL-18, whereas cells infected with T3SS/flagellin-deficient strain Δ*prgJ*Δ*fljB*Δ*fliC* did not secrete cytokines at 2 h (Fig. 8B, 8C). These cytokine data were consistent with the cell viability data. Infection with T3SS/flagellin-deficient strain *ΔprgJΔfljBΔfliC* at MOI 50 induced only low levels of cell death at 24 h postinfection, leading to high levels of cytokine secretion (Supplemental Fig. 4). We hypothesized that cytokine production at 24 h was likely to be due to either canonical or noncanonical NLRP3 inflammasome activation, but we would expect this to be accompanied by profound cell death. Infection of hMDM cells with *S.* Typhimurium in the presence of the NLRP3 inhibitor MCC950, however, had no effect on cytokine release or cell viability, suggesting that a NLRC4- or NLRP3-independent pathway may be operating in *S.* Typhimurium–infected hMDM (Fig. 8A–C).

In conclusion, hMDM displayed cell death and cytokine secretion in response to wild-type and flagellin-deficient *S.* Typhimurium strain Δ*fljB*Δ*fliC* but not in response to the T3SS/flagellin-deficient strain Δ*prgJ*Δ*fljB*Δ*fliC*.

## Discussion

In this study, we show that inflammasome activation in human macrophage models involves multiple inflammasome receptors and different ligands in response to infection with live *S.* Typhimurium. Flagellin can trigger both NAIP/NLRC4 and the canonical NLRP3 inflammasome in THP-1 cells, but NLRP3 activation is only apparent in NAIP- or NLRC4-deficient cells. This is harder to verify in hMDM because of the high levels of NAIP, which may act as a binding “sink” for flagellin such that it is not free to trigger NLRP3. Our data suggest that flagellin can activate two different NLRs, namely NAIP/NLRC4 and NLRP3, and we hypothesize that NLRP3 can act as a safety net, triggering inflammasome activation under conditions in which NAIP/NLRC4 is poorly activated.

Multiple groups have reported the dual activation of NAIP/NLRC4 and NLRP3 upon *S.* Typhimurium infection in human and murine macrophages (4–7). The activation of the NAIP/NLRC4 pathway by bacterial protein ligands, in particular flagellin, and the relative contribution of the different ligands to inflammasome activation during bacterial infection are better characterized in murine macrophages than in human cells. Mouse macrophages respond very efficiently to flagellins from many different pathogens, including *Legionella pneumophila*, *S.* Typhimurium, *Pseudomonas aeruginosa*, or *B. subtilis* delivered by infection, transfection, or a *B. anthracis* toxin-based delivery method (9, 11, 14, 33–38). Flagellin sensing appears to be important in mice because they express two Naip proteins, Naip 5 and 6, dedicated to this function (9, 12, 13). Humans only have one NAIP protein that can sense the bacterial ligands flagellin and T3SS needle and rod proteins (9, 18–20). Interestingly, human NAIP shares the highest homology with the needle-sensing murine Naip1. In this study, PrgI triggered a seemingly more potent inflammasome response than flagellin in both THP-1 cells and hMDM (Fig. 3), although we cannot rule out that this apparent difference in inflammasome triggering is due to a higher transfection efficiency of the smaller PrgI protein in comparison with flagellin. hMDM express higher levels of NAIP than THP-1 cells, which could make them more sensitive to ligands such as PrgI. Increasing NAIP levels in U937 cells led to enhanced cell death in response to *S.* Typhimurium infection (20). In our study, flagellin-deficient *S.* Typhimurium SL1344 *ΔfljBΔfliC* triggered inflammasome activation in hMDM but not THP-1 cells. This could indicate that NAIP expression in THP-1 cells may be insufficient to sense the T3SS proteins in the context of bacterial infection, assuming equal levels of infection in both macrophage models. Our findings contrast with those from an earlier study, which proposed that inflammasome activation is dependent on flagellin in hMDM (20). These observations can be reconciled by the delayed kinetics of cell death and cytokine secretion induced by the flagellin-deficient *Salmonella* strain lacking Δ*fljB*Δ*fliC*, as in the previous study, inflammasome activation was assessed only at 1 h postinfection. In the absence of T3SS/flagellin, *S.* Typhimurium–infected macrophages secreted IL-1β and IL-18 without marked cell death at 24 h postinfection (Fig. 4A–C, Supplemental Fig. 2). This was independent of NLRP3 as MCC950 could neither inhibit cytokine secretion nor increase cell viability in hMDM at this time point. Using a SPI1-deficient *S.* Typhimurium strain, Bierschenk et al. (4) observed a similar phenotype in primary hMDM. Alternative enzymes to caspase-1, including caspase-8, are known to be able to cleave pro–IL-1β in an inflammasome-dependent and -independent manner (39). We speculate that upon infection of hMDM with T3SS/flagellin-deficient *S.* Typhimurium, IL-1β and IL-18 are processed in a caspase-8–dependent manner and released in a GSDMD-independent way.

Dissecting the inflammasome pathways involved, we show that THP-1 cells activate NLRP3 in the absence of NAIP or NLRC4 upon transfection of *S.* Typhimurium flagellin. In contrast, in murine macrophages, NLRP3 activation in response to flagellin is fully dependent on NLRC4 (7). In THP-1 cells, it is likely that flagellin activates human NAIP by binding in a similar fashion as reported for the interaction between flagellin–NAIP5 (12, 17). It remains unclear, however, whether activation of NAIP and NLRP3 occur simultaneously in activated cells.

We established that flagellin activates NLRP3 in a NAIP-independent manner and that its activation occurs through the canonical pathway because NLRP3 activation is independent of upstream caspase activation, indicating that caspase-4/-5 are not required for this response (Fig. 5A, 5B). These data rule out the possibility that NLRP3 activation by *S.* Typhimurium flagellin was mediated by potential LPS contamination of this protein to drive noncanonical inflammasome activation. This is further supported by our data showing that flagellin derived from the Gram-positive bacterium *B. subtilis* also triggers NLRP3 activation (Fig. 6A). NLRP3 activation cannot be a nonspecific mechanism induced by protein transfection into the cytosol because transfection of PrgI only activates NAIP/NLRC4. Both *Salmonella*- and *B. subtilis*–derived flagellin triggered the NLRP3 inflammasome in a ROS- and cathepsin-dependent manner (Fig. 6B). Cathepsin B is required for efficient NLRP3 activation in response to many well-known NLRP3 activators, and it is thought to interact directly with NLRP3 in THP-1 cells (40). Others have reported that transfected flagellin induces cathepsin release in murine macrophages (36). Together with our data, these observations provide a potential explanation as to how intracellular flagellin activates the NLRP3 inflammasome.

Flagellin-mediated activation of NLRP3 was not apparent in hMDM, but there are two potential reasons for this. In THP-1 cells, KO of *NAIP* or *NLRC4* was required to fully reveal this pathway. We cannot, currently, disrupt *NAIP* or *NLRC4* from hMDM, and flagellin-mediated activation of NAIP/NLRC4 likely masks any role for NLRP3. In hMDM, inflammasome activation in response to *S.* Typhimurium is not reduced by the NLRP3 inhibitor MCC950 alone (Fig. 8), similar to data from Bierschenk et al. (4). We also hypothesize that the high levels of NAIP in hMDMs, in comparison with THP-1 cells, may lead to efficient flagellin binding, reducing the possibility of this protein being available to activate NLRP3. It is possible, therefore, that only by removal of NAIP expression in hMDM will flagellin activation of NLRP3 activation be revealed. Flagellin-mediated inflammasome activation is reduced in hMDM upon NAIP knockdown (18), suggesting that there are subtle differences in NLRP3 activation by flagellin. It is tempting to speculate that NLRP3 activation can complement inefficient NAIP/NLRC4 activation in human macrophages under certain conditions, for example in sepsis when LPS levels are high, as our mRNA expression data suggests NLRC4 expression is lowered under these conditions.

Our study contributes to the understanding of the complex interplay between NAIP/NLRC4 and NLRP3 inflammasome receptors in human macrophages. Using inflammasome-deficient macrophages, we identified that flagellin can trigger NLRP3, whereby this activation may act as a safety net to preserve inflammasome activation in the face of suboptimal NAIP/NLRC4 activation. These complexities in the human NAIP/NLRC4 pathway contrast with the murine pathway, whereby multiple ligand-specific Naip molecules facilitate highly efficient activation to all ligands.

## Supplementary Material

Data Supplement
