# Supplementary material for: Salmonella Flagellin Activates NAIP/NLRC4 and Canonical NLRP3 Inflammasomes in Human Macrophages
Source: J Immunol. 2020 Dec 30;206(3):631–40. doi: 10.4049/jimmunol.2000382 (PMC7812056; doi:10.4049/jimmunol.2000382)
Supplement: Data Supplement [file JI_2000382.zip › JI_2000382_Supplemental_Figures_1.pdf]

Supplementary Figure 1 Characterization of THP-1 KO cells

A *NLRC4* KO clones

```
NLRC4 WT allele      ACGAAGACTCAGCAGTTTATTGACGTCTCATGAGCCAGAGGAGGTGACCAAGGGGAATGGTTACTTGCAGAAAATGGTTT

#1 KO allele 1       ACGAAGACTCAGCAGTTTATTGACGTCTCATGA--------------CCAAGGGGAATGGTTACTTGCAGAAAATGGTTT 14nt deletion
#1 KO allele 2       ACGAAGACTCAGCAGTTTATTGACGTCTCATGAGCCAG-----GTGACCAAGGGGAATGGTTACTTGCAGAAAATGGTTT  5nt deletion
#2 KO allele 1/2     ACGAAGACTCAGCAGTTTATTGACGTCTCATGAGCCAG--GAGGTGACCAAGGGGAATGGTTACTTGCAGAAAATGGTTT  2nt deletion
NLRC4 WT allele      GAACCTGTCCTGTGGAGGAAGGACCAACACCATCACCGCGTGGAGCAGCTGACCCTGAATGGCCTCCTGCAGGCTCTTCA

#3 KO allele 1       GAACCTGTCCTGTGGAGGA----------------CAGCGTGGAGCAGCTGACCCTGAATGGCCTCCTGCAGGCTCTTCA 16nt deletion
#3 KO allele 2       GAACCTGTCCTGTGGAGGAAGGACCAACACCATCAC-GCGTGGAGCAGCTGACCCTGAATGGCCTCCTGCAGGCTCTTCA  1nt deletion
#4 KO allele 1/2     GAACCTGTCCTGTGGAGGAAGGACCAACACCATCAC-GCGTGGAGCAGCTGACCCTGAATGGCCTCCTGCAGGCTCTTCA  1nt deletion
```

B *NLRP3* KO clones

```
NLRP3 WT allele      GACATCCAAGACCACCACCGCGGT-GTACGTCTTCTTCCTTTCCAGTTTGCTGCAGCCCCGGGGAGGGAGCCAGGAGCACG

#1/2 KO allele 1     GACATCCAAGACCACCACCGCGG--GTACGTCTTCTTCCTTTCCAGTTTGCTGCAGCCCCGGGGAGGGAGCCAGGAGCACG  1nt deletion
#1/2 KO allele 2     GACATCCAAGACCACCACCGCGGTTGTACGTCTTCTTCCTTTCCAGTTTGCTGCAGCCCCGGGGAGGGAGCCAGGAGCACG  1nt insertion
#3 KO allele 1/2     GACATCCAAGACCACCACCGCGGTTGTACGTCTTCTTCCTTTCCAGTTTGCTGCAGCCCCGGGGAGGGAGCCAGGAGCACG  1nt insertion
#4 KO allele 1       GACATCCAAGACCACCACCGC------------------------------TGCAGCCCCGGGGAGGGAGCCAGGAGCACG 29nt deletion
#4 KO allele 2       GACATCCAAGACCACCACCGCGGT---ACGTCTTCTTCCTTTCCAGTTTGCTGCAGCCCCGGGGAGGGAGCCAGGAGCACG  2nt deletion
```

C *NLRC4/NLRP3* DKO clones

```
NLRC4 WT allele      ACGAAGACTCAGCAGTTTATTGACGTCTCATGAGCCAGAGGAGGTGACCAAGGGGAATGGTTACTTGCAGAAAATGGTTT

#1/2 KO allele 1     ACGAAGACTCAGCAGTTTATTGACGTCTCATGAGCCA-AGGAGGTGACCAAGGGGAATGGTTACTTGCAGAAAATGGTTT  1nt deletion
#1/2 KO allele 2     ACGAAGACTCAGCAGTTTATTGACGTCTCATGAGCCAG--GAGGTGACCAAGGGGAATGGTTACTTGCAGAAAATGGTTT  2nt deletion
#3 KO allele 1/2     ACGAAGACTCAGCAGTTTATTGACGTCTCATGAGCCA--CCTCATGACCAAGGGGAATGGTTACTTGCAGAAAATGGTTT  2nt deletion
#4 KO allele 1       ACGAAGACTCAGCAGTTTATTGACGTCTCATGA-----AGGAGGTGACCAAGGGGAATGGTTACTTGCAGAAAATGGTTT  5nt deletion
#4 KO allele 2       ACGAAGACTCAGCAGTTTATTGACGTCTCATGAGCCAG--GAGGTGACCAAGGGGAATGGTTACTTGCAGAAAATGGTTT  2nt deletion

NLRP3 WT allele      GACATCCAAGACCACCACCGCGGT-GTACGTCTTCTTCCTTTCCAGTTTGCTGCAGCCCCGGGGAGGGAGCCAGGAGCACG

#1/2 KO allele 1     GACATCCAAGACCACCACCGCGG--GTACGTCTTCTTCCTTTCCAGTTTGCTGCAGCCCCGGGGAGGGAGCCAGGAGCACG  1nt deletion
#1 KO allele 2       GACATCCAAGACCACCACCGCGGT---ACGTCTTCTTCCTTTCCAGTTTGCTGCAGCCCCGGGGAGGGAGCCAGGAGCACG  2nt deletion
#2 KO allele 2       GACATCCAAGACCACCACCGCGGTTGTACGTCTTCTTCCTTTCCAGTTTGCTGCAGCCCCGGGGAGGGAGCCAGGAGCACG  1nt insertion
#3/4 KO allele 1/2   GACATCCAAGACCACCACCGCGGTTGTACGTCTTCTTCCTTTCCAGTTTGCTGCAGCCCCGGGGAGGGAGCCAGGAGCACG  1nt insertion
```

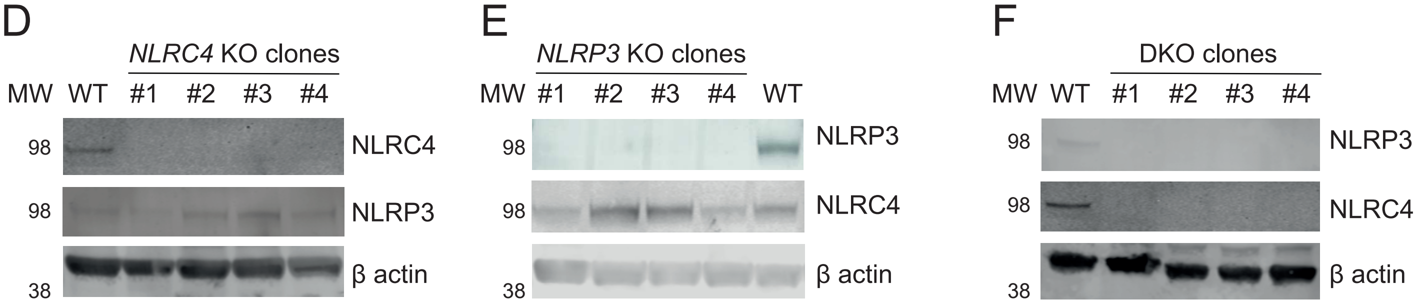

G *NAIP* KO clones

```
NAIP WT allele       GTACCAAGAAGAGGAGGCTAGACTTGCGTCCTTCAGGAA-CTGGCCATTTTATGTCCAAGGGATATCCCCTTGTGTGCTCT

#1 KO allele 1/2     GTACCAAGAAGAGGAGGCTAGACTTGCATCCTTCAG-AA-CTGGCCATTTTATGTCCAAGGGATATCCCCTTGTGTGCTCT  1nt deletion

#2 KO allele 1/2     GTACCAAGAAGAGGAGGCTAGACTTGCATCCTTCT--AA-CTGGCCATTTTATGTCCAAGGGATATCCCCTTGTGTGCTCT  2nt deletion

#3 KO allele 1       GTACCAAGAAGAGGAGGCTAGACTTGCGTCCTT---------------TTTATGTCCAAGGGATATCCCCTTGTGTGCTCT 14nt deletion
#3 KO allele 2       GTACCAAGAAGAGGAGGCTAGACTTGCATCCTTCA--AA-CTGGCCATTTTATGTCCAAGGGATATCCCCTTGTGTGCTCT  2nt deletion

#4 KO allele 1       GTACCAAGAAGAGGAGGCTAGACTTGCATCCTT----AT-CTGGCCATTTTATGTCCAAGGGATATCCCCTTGTGTGCTCT  4nt deletion
#4 KO allele 2       GTACCAAGAAGAGGAGGCTAGACTTGCGTCCTTCAGGAAACTGGCCATTTTATGTCCAAGGGATATCCCCTTGTGTGCTCT  1nt insertion
```

Supplementary figure 1 Characterization of THP-1 KO cells. Genotypes (A-C, G) and/or Western blot analysis (D-F) of THP-1 *NLRC4* KO (A, D), *NLRP3* KO (B, E), *NLRP3/NLRC4* DKO (C, F), *NAIP* KO (G) clones. Four validated KO clones are shown. Deletions are indicated by -, insertions are shown in italics and bases differing from consensus sequence are shown in bold italics.

Supplementary Figure 2 Inflammasome activation in THP-1 cells requires both NLRP3 and NLRC4 in response to infection by *Salmonella* and is flagellin -dependent

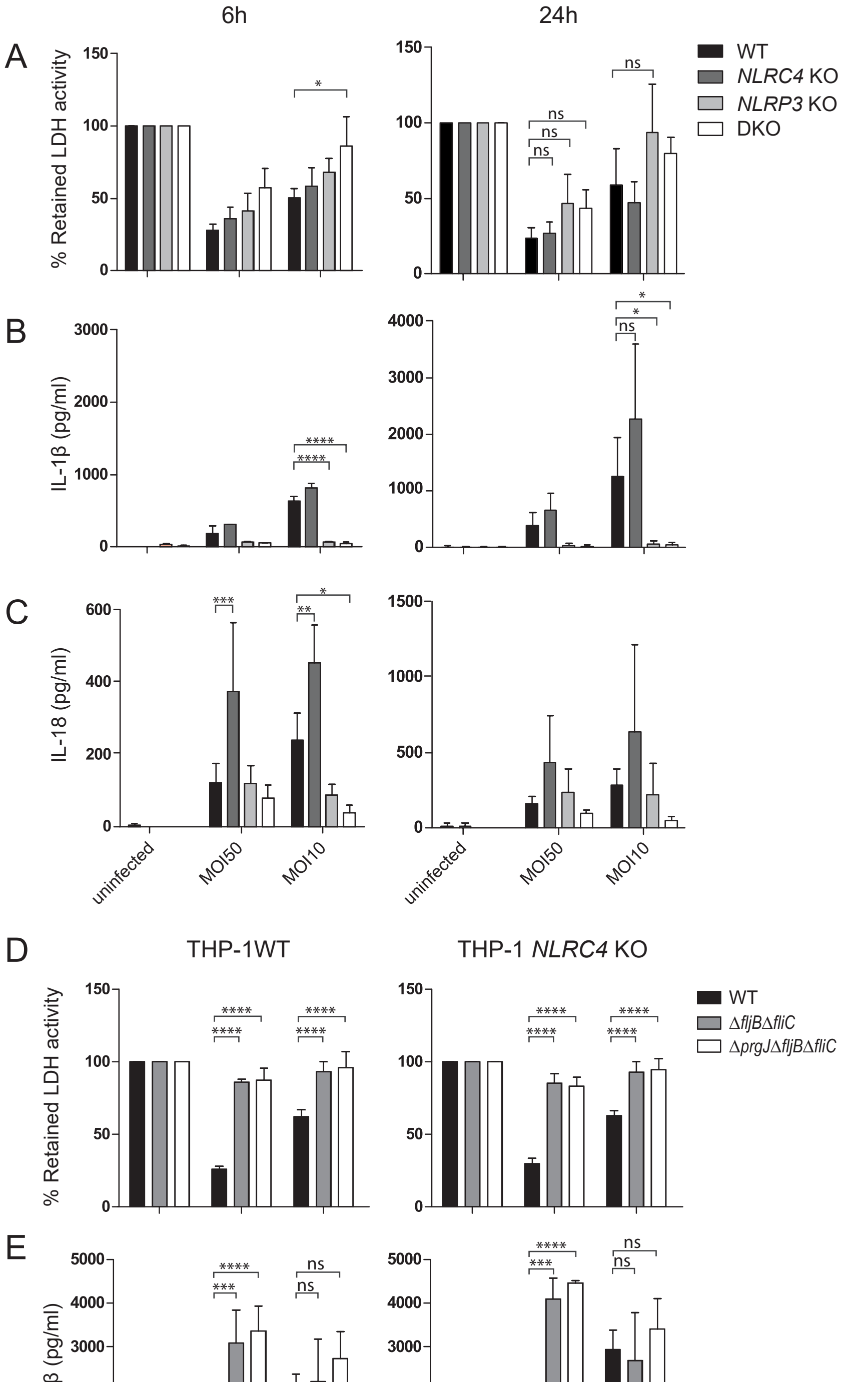

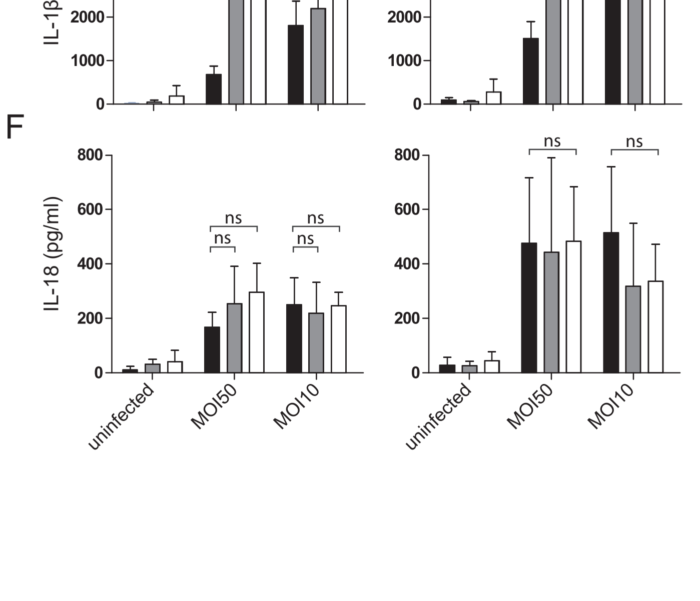

Supplementary figure 2 Inflammasome activation in THP-1 cells requires both NLRP3 and NLRC4 in response to infection by S. Typhimurium and is flagellin-dependent. A-C) THP-1 wild-type, *NLRC4* KO, *NLRP3* KO, or *NLRC4/NLRP3* 'DKO' cells were infected with wild-type S. Typhimurium at MOI50 or 10. Cell viability (A) and cytokine secretion (B, C) were assessed at 6h and 24h post-infection. D-F) THP-1 wild-type and *NLRC4* KO cells were infected with wild-type, flagellin-deficient (*ΔfljBΔfliC*) or flagellin/T3SS-deficient (*ΔprgJΔfljBΔfliC*) S. Typhimurium at MOI50 or 10. Cell viability (D) and cytokine secretion (E, F) were assessed at 24h post-infection. Mean with SD of two (B, IL-1β) or three (A, C-F; cell viability, IL-18) independent experiments are shown.

Statistical analysis was performed using a 2-way ANOVA followed by a Bonferroni post-test; * P<0.05, ** p< 0.01, *** p< 0.001, **** p< 0.001

Supplementary Figure 3 Recombinant PrgI triggers the NAIP/NLRC4 inflammasome, whereas flagellin activates the NAIP/NLRC4 and the NLRP3 inflammasome in THP-1 cells

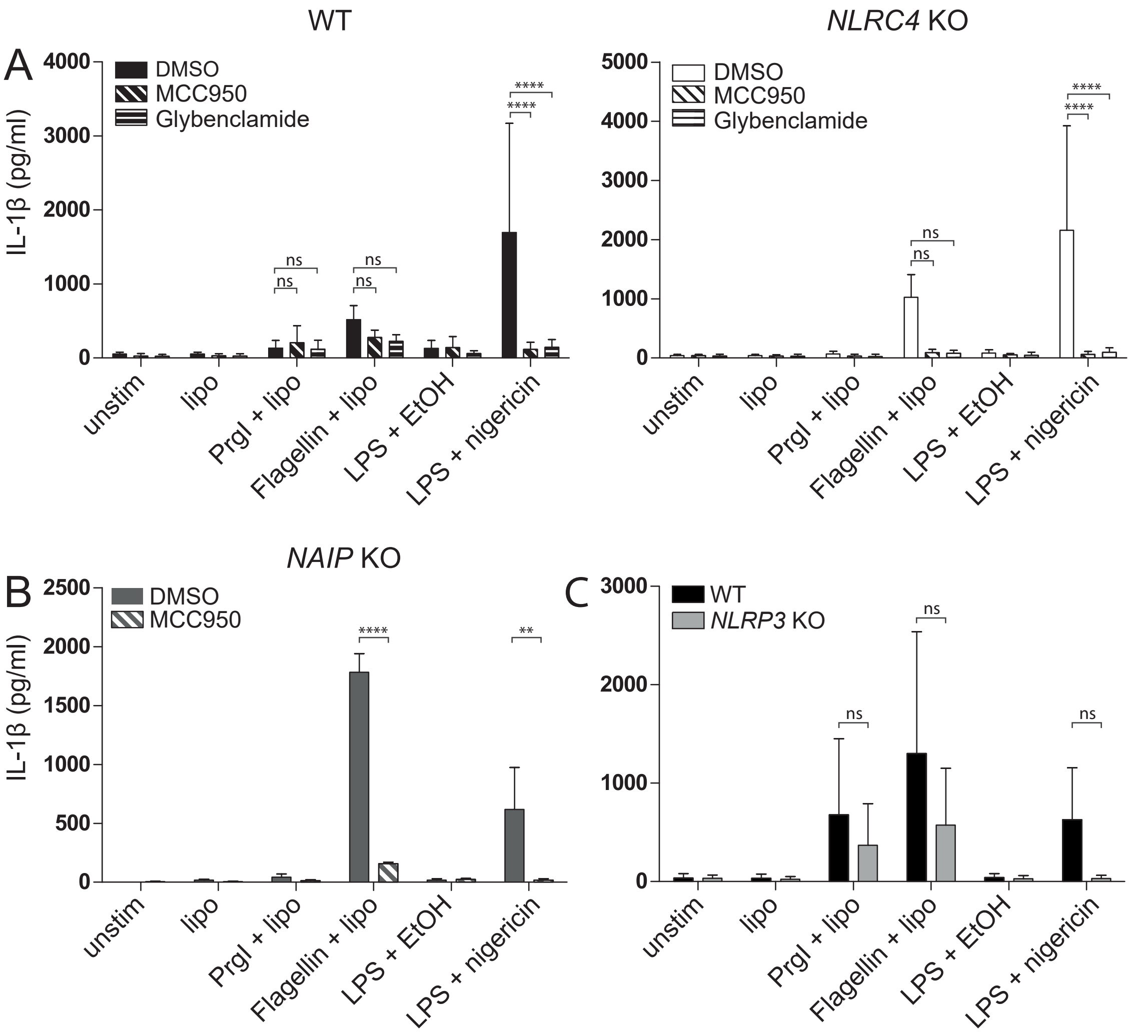

Supplementary figure 3 Recombinant PrgI triggers the NAIP/NLRC4 inflammasome, whereas flagellin activates the NAIP/NLRC4 and the NLRP3 inflammasome in THP-1 cells. Parental wild-type THP-1 (A, C), *NLRC4* KO (A), *NAIP* KO (B) or *NLRP3* KO (C) cells were transfected with PrgI or flagellin or stimulated with LPS+ Nigericin in the presence of NLRP3 inhibitors MCC950 (A, B) or glybenclamide (B). IL-1β release was determined at 4h post-stimulation. Mean with SD of at least three individual experiments is displayed. Statistical analysis was performed using 2-way ANOVA followed by a Bonferroni post-test, * $p<0.05$, ** $p< 0.01$, *** $p< 0.001$, **** $p< 0.0001$

Supplementary Figure 4 In hMDM, flagellin-deficient *S.* Typhimurium is sufficient to trigger inflammasome activation, which is NLRP3 independent

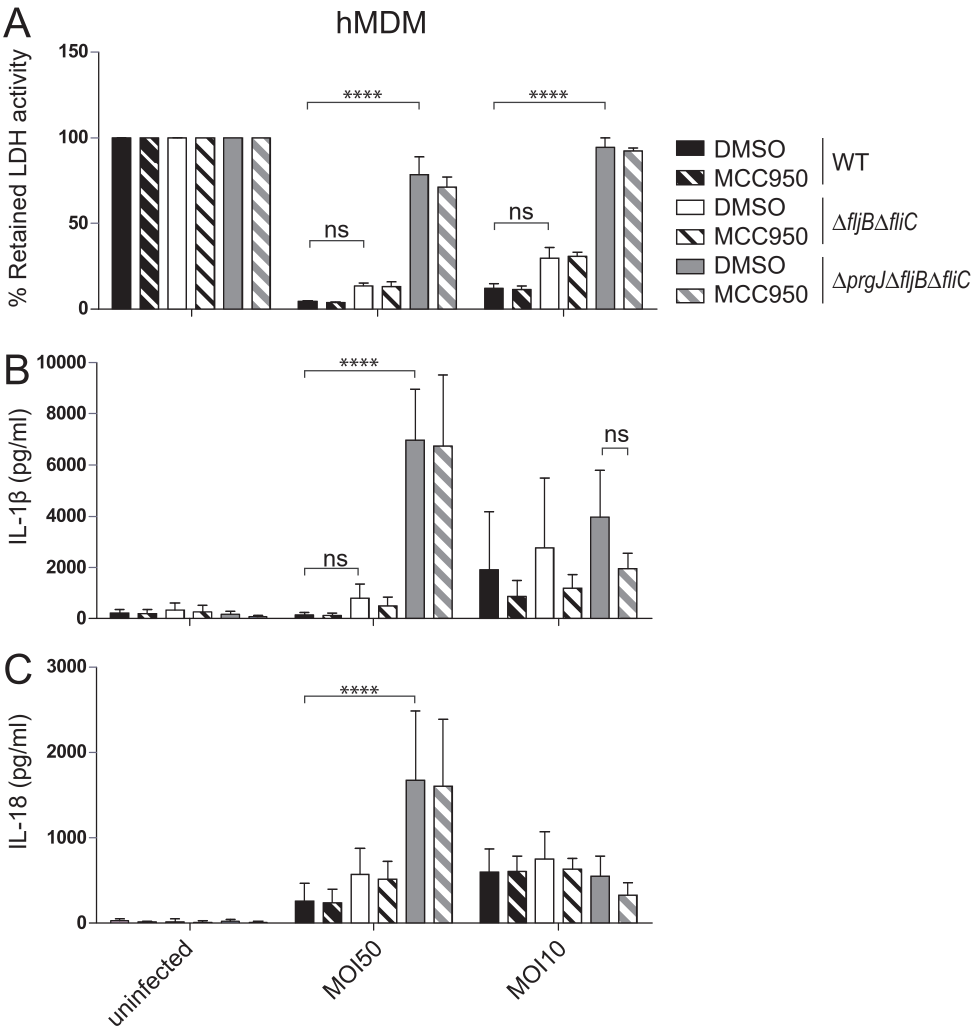

Supplementary figure 4 In hMDM, flagellin-deficient *S.* Typhimurium is sufficient to trigger inflammasome activation, which is NLRP3 independent. hMDM were infected with S. Typhimurium at MOI50 or 10. Cell viability (A) and cytokine secretion (B, C) were assessed at 24h post-infection. Mean with SD of three different donors is displayed. Two-way ANOVA followed by a Bonferroni post-test; **** $p < 0.0001$
